# Contributions to the phylogeny of *Ixodes* (*Pholeoixodes*) *canisuga*, *I.* (*Ph.*) *kaiseri*, *I.* (*Ph.*) *hexagonus* and a simple pictorial key for the identification of their females

**DOI:** 10.1186/s13071-017-2424-x

**Published:** 2017-11-03

**Authors:** Sándor Hornok, Attila D. Sándor, Relja Beck, Róbert Farkas, Lorenza Beati, Jenő Kontschán, Nóra Takács, Gábor Földvári, Cornelia Silaghi, Elisabeth Meyer-Kayser, Adnan Hodžić, Snežana Tomanović, Swaid Abdullah, Richard Wall, Agustín Estrada-Peña, Georg Gerhard Duscher, Olivier Plantard

**Affiliations:** 1 Department of Parasitology and Zoology, University of Veterinary Medicine, Budapest, Hungary; 2 Department of Parasitology and Parasitic Diseases, University of Agricultural Sciences and Veterinary Medicine, Cluj-Napoca, Romania; 3 Department for Bacteriology and Parasitology, Croatian Veterinary Institute, Zagreb, Croatia; 4 U.S. National Tick Collection, Institute for Coastal Plain Science, Georgia Southern University, Statesboro, GA USA; 5 Plant Protection Institute, Centre for Agricultural Research, Hungarian Academy of Sciences, Budapest, Hungary; 6 National Centre for Vector Entomology, Institute of Parasitology, Vetsuisse Faculty, University of Zurich, Zurich, Switzerland; 7 State Office for Consumer Protection (TLV), Bad Langensalza, Germany; 8 Department of Pathobiology, Institute of Parasitology, University of Veterinary Medicine Vienna, Vienna, Austria; 9 Department for Medical Entomology, Institute for Medical Research, University of Belgrade, Belgrade, Serbia; 10 Veterinary Parasitology and Ecology Group, School of Biological Sciences, Life Sciences Building, University of Bristol, Bristol, UK; 11 Department of Animal Health, Faculty of Veterinary Medicine, Zaragoza, Spain; 12 BIOEPAR, INRA, Oniris, Nantes, France

**Keywords:** Carnivora, Erinaceidae, *Ixodes crenulatus*, *Ixodes rugicollis*

## Abstract

**Background:**

In Europe, hard ticks of the subgenus *Pholeoixodes* (Ixodidae: *Ixodes*) are usually associated with burrow-dwelling mammals and terrestrial birds. Reports of *Pholeoixodes* spp. from carnivores are frequently contradictory, and their identification is not based on key diagnostic characters. Therefore, the aims of the present study were to identify ticks collected from dogs, foxes and badgers in several European countries, and to reassess their systematic status with molecular analyses using two mitochondrial markers.

**Results:**

Between 2003 and 2017, 144 *Pholeoixodes* spp. ticks were collected in nine European countries. From accurate descriptions and comparison with type-materials, a simple illustrated identification key was compiled for adult females, by focusing on the shape of the anterior surface of basis capituli. Based on this key, 71 female ticks were identified as *I. canisuga*, 21 as *I. kaiseri* and 21 as *I. hexagonus*. DNA was extracted from these 113 female ticks, and from further 31 specimens. Fragments of two mitochondrial genes, *cox*1 (cytochrome *c* oxidase subunit 1) and 16S rRNA, were amplified and sequenced. *Ixodes kaiseri* had nine unique *cox*1 haplotypes, which showed 99.2–100% sequence identity, whereas *I. canisuga* and *I. hexagonus* had eleven and five *cox*1 haplotypes, respectively, with 99.5–100% sequence identity. The distribution of *cox*1 haplotypes reflected a geographical pattern. *Pholeoixodes* spp. ticks had fewer 16S rRNA haplotypes, with a lower degree of intraspecific divergence (99.5–100% sequence identity) and no geographical clustering. Phylogenetic analyses were in agreement with morphology: *I. kaiseri* and *I. hexagonus* (with the similar shape of the anterior surface of basis capituli) were genetically more closely related to each other than to *I. canisuga*. Phylogenetic analyses also showed that the subgenus *Eschatocephalus* (bat ticks) clustered within the subgenus *Pholeoixodes*.

**Conclusions:**

A simple, illustrated identification key is provided for female *Pholeoixodes* ticks of carnivores (including *I. hexagonus* and *I. rugicollis*) to prevent future misidentification of these species. It is also shown that *I. kaiseri* is more widespread in Europe than previously thought. Phylogenetic analyses suggest that the subgenus *Pholeoixodes* is not monophyletic: either the subgenus *Eschatocephalus* should be included in *Pholeoixodes*, or the latter subgenus should be divided, which is a task for future studies.

## Background

Hard ticks (Acari: Ixodidae) are regarded as the most important vectors of pathogens [1]. Among them, the genus *Ixodes* Latreille, 1795 contains the highest number of species, exceeding 244 [2]. Traditionally, this genus was subdivided into subgenera, eight of which have representatives in the western Palaearctic [3]. The subgenus *Pholeoixodes* was erected [4] based on common morphological and ecological features of its members. For instance, the females of this subgenus have relatively short palps, there are no auriculae on the ventral basis capituli, and the first tarsi show a subapical dorsal hump [5]. *Pholeoixodes* species are usually associated with burrow-dwelling mammals, as well as terrestrial birds that nest in cavities (tree holes or burrows). Species of this subgenus usually feed on mammals, particularly carnivores (mainly Canidae, Mustelidae) and hedgehogs (Erinaceidae), in the western Palaearctic including *I. canisuga* Johnston, 1849, *I. kaiseri* Arthur, 1957, *I. crenulatus* Koch, 1844, *I. hexagonus* Leach, 1815 and *I. rugicollis* Schulze & Schlottke, 1929.

Revised extensively by Babos [6], the systematics of the subgenus *Pholeoixodes* has yet to be fully understood. In particular, problems with identification and nomenclature led to the incorrect definition of geographical ranges. For instance, *I. crenulatus* was considered to occur in western, central and eastern Europe [7]. However, recent studies on ticks from carnivores confirmed its presence in Romania [8], but not in central and western Europe [9–11]. Furthermore, the validity of *I. crenulatus* was questioned because of its uninformative description that often results in it being misidentified as *I. hexagonus* [12]. Also, based on the morphological similarities of *I. crenulatus* and *I. kaiseri*, their synonymization was proposed [13], and later rejected [14].

*Ixodes hexagonus* and *I. canisuga* are common species on carnivores in the western Palaearctic [3, 11, 15–17]. While *I. hexagonus* can also be found in eastern Europe [8, 9, 14], *I. canisuga* occurs predominantly in western and central Europe [9, 18, 19]. In regions where they are sympatric, the classical morphological approach to distinguishing females of these two species is the observation of the internal spur on the first coxae, which is present in *I. hexagonus*, but absent in *I. canisuga* [20]. However, *I. hexagonus* appears to be sometimes mistakenly identified as *I. canisuga*, as concluded from identical GenBank sequences (e.g. [21]: *I. canisuga* [JF928508], *I. hexagonus* [AF001400]). Furthermore, both species may be easily mistaken with *I. crenulatus* [12, 22].

**Fig. 1:**
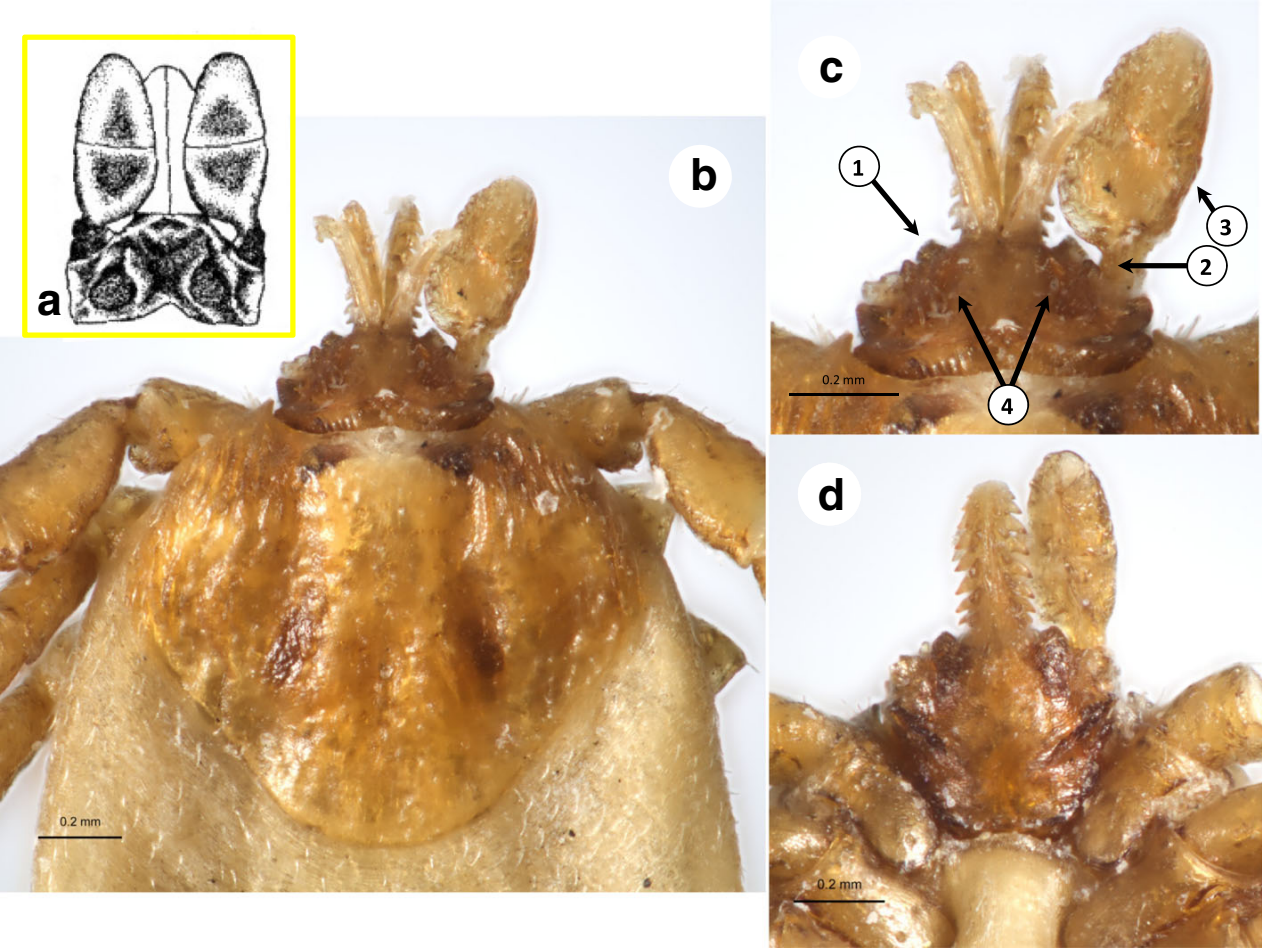
Key features of *Ixodes rugicollis* females. **a** Original drawing of the gnathosoma by Schulze & Schlottke [23]). **b**-**d** Female syntype (USNTC): **b** scutum with “rugose” (wrinkled) surface, and basis capituli; **c** basis capituli, dorsal surface, enlarged; **d** basis capituli, ventral aspect. Numbered arrows indicate (in the order of presentation in the key): (1) pronounced, pointed frontal bump near the hypostome basis; (2) “stalked” palp; (3) curved (convex) lateral edge of palp; (4) broad space between inconspicuous, small porose areas

Another controversy regarding the systematics and morphology of *Pholeoixodes* species is related to *I. rugicollis*. In the original drawing of the gnathosoma of *I. rugicollis* [23] (Fig. 1a), there was no indication of two frontal bumps on the anterior surface of basis capituli (between the basis of the hypostome and the palps). Later this species was even depicted without frontal bumps [7]. Furthermore, the drawings of the gnathosoma of *I. rugicollis* in its original description [23] and redescription [24] do not show broad separation of the porose areas, unlike what was reported in an electron microscopic study [25]. *Ixodes rugicollis* was also synonymized with *I. cornutus* [26], the porose areas of which are relatively close to each other [14].

In light of the above uncertainties, when contradictions arise with the traditional morphology-based identification of ticks, molecular comparison of representative specimens may provide additional and important clues to solve problems. Recently, North American members of the subgenus *Pholeoixodes* have been included in such molecular analyses [27]. However, no phylogenetic study has been performed on *Pholeoixodes* tick species collected from carnivores in Europe. Therefore, the aims of the present study were to morphologically identify female ticks collected from dogs, foxes and badgers in nine European countries, with subsequent molecular analyses using two mitochondrial markers. The study also aimed to clarify the taxonomic status and phylogenetic relationships of some *Pholeoixodes* species.

## Methods

### Sample collection and tick identification

Ticks were collected between 2003 and 2017. Specimens from Germany were stored frozen, and others were stored in 96% ethanol. Female ticks (except those from Serbia, from which the DNA was extracted previously) were examined morphologically with a VHX-5000 digital microscope (Keyence Co., Osaka, Japan). For species identification of females within *Pholeoixodes*, the following literature sources and type-materials were used: *I. hexagonus* [14], *I. canisuga* (neotypes loaned by the Natural History Museum, London, UK [NHM], accession numbers NHMUK 010305616–8: collected from dogs, UK), *I. kaiseri* ([14, 28]; paratype deposited in the United States National Ticks Collection [USNTC], accession number USNMENT00859298; and paratype loaned by NHM, accession number 1957.1.28.1: collected from fox, Egypt), *I. crenulatus* [14], *I. rugicollis* (syntype deposited in the USNTC, accession number USNMENT00865840: collected from pine marten, Germany). Adult ixodid ticks are usually easier to identify at the species level than immature stages, therefore morphological comparisons focused on adult female specimens. Nymphs were identified molecularly with PCR and sequencing two mitochondrial markers, which were compared to those of female ticks.

### Molecular and phylogenetic analyses

DNA was extracted from the ticks (from part of the idiosoma and/or legs) individually with the QIAamp DNA Mini Kit (Qiagen, Hilden, Germany) according to the manufacturer’s instructions, including an overnight digestion in tissue lysis buffer and Proteinase-K at 56 °C. The cytochrome *c* oxidase subunit I (*cox*1) gene was chosen as the primary target for molecular analysis, on account of its suitability as a DNA-barcode sequence for tick species identification [29]. The PCR (modified from [30]) amplifies an approximately 710 bp long fragment of the *cox*1 gene. The primers HCO2198 (5′-TAA ACT TCA GGG TGA CCA AAA AAT CA-3′) and LCO1490 (5′-GGT CAA CAA ATC ATA AAG ATA TTG G-3′) were used in a reaction volume of 25 μl, containing 1 U (0.2 μl) HotStar*Taq* Plus DNA polymerase (Qiagen), 2.5 μl 10× CoralLoad Reaction buffer (including 15 mM MgCl_2_), 0.5 μl PCR nucleotide Mix (0.2 mM each), 0.5 μl (1 μM final concentration) of each primer, 15.8 μl ddH_2_O and 5 μl template DNA. For amplification, an initial denaturation step at 95 °C for 5 min was followed by 40 cycles of denaturation at 94 °C for 40 s, annealing at 48 °C for 1 min and extension at 72 °C for 1 min. Final extension was performed at 72 °C for 10 min.

To complement the results obtained with the *cox*1 gene, all samples that showed different *cox*1 haplotype within a country, were also tested for another mitochondrial marker. This PCR amplifies an approximately 460 bp fragment of the 16S rRNA gene of Ixodidae [31], with the primers 16S + 1 (5′-CTG CTC AAT GAT TTT TTA AAT TGC TGT GG-3′) and 16S-1 (5′-CCG GTC TGA ACT CAG ATC AAG T-3′). Other reaction components and cycling conditions were the same as above, except for annealing at 51 °C.

PCR products were visualized in a 1.5% agarose gel. Purification and sequencing were done by Biomi Inc. (Gödöllő, Hungary). If identical sequences were found in a country, only one representative sequence was submitted to GenBank (accession numbers for the *cox*1 gene: KY962011–KY962051; for the 16S rRNA gene: KY962052–KY962077). Positions of nucleotide differences according to haplotypes are provided in Tables 1, 2.

**Table 1:** Data of samples used in this study, and results of *cox*1 sequence analyses

Region	Country	Locality or region of collection	Sample origin	*Ixodes* species (number)	*cox*1 haplotype name^a^ (number if >1)	GenBank accession nos.
Western Europe	UK	Bridgewater	dog	*I. canisuga* (2F)	H, J	KY962048, KY962047
		Glantwymyn	dog	*I. canisuga* (2F)	H (2×)	KY962048
		Plymtree	dog	*I. canisuga* (2F)	H (2×)	KY962048
		Whissonsett	dog	*I. canisuga* (2F)	H (2×)	KY962048
	France	Bernay	fox burrow	*I. canisuga* (1F, 1N)	H (2×)	KY962049
		Nancy	badger	*I. canisuga* (1F, 3N)	A (2×), F, H	KY962050, KY962051, KY962049
		Nantes	badger	*I. canisuga* (1N)	H	KY962049
		Carquefou	badger	*I. canisuga* (1F, 4N)	H (5×)	KY962049
Central Europe	Germany	Thuringia	red fox	*I. kaiseri* (4F)	M (3×), O	KY962042, KY962043
		Thuringia	red fox	*I. canisuga* (6F)	A (4×), H (2×)	KY962044, KY962045
		Thuringia	red fox	*I. hexagonus* (3F)	X (3×)	KY962046
	Austria	Burgenland	dog	*I. hexagonus* (2N)	X (2×)	KY962019
	Hungary	Budapest	dog	*I. kaiseri* (8F)	L (5×), S, P (2×)	KY962011, KY962014, KY962015
		Budapest	dog	*I. canisuga* (2F)	A, B	KY962013, KY962012
South-eastern Europe	Croatia	Jastrebarsko	red fox	*I. hexagonus* (18F)	U (4×), V (12×), W, Y	KY962036, KY962035, KY962034, KY962041
		Jastrebarsko	red fox	*I. canisuga* (35F)	A (3×), E, G (23×), I (8×)	KY962040, KY962039, KY962037, KY962038
	Bosnia and Herzegovina	Srebrenik, Zvornik	red fox	*I. canisuga* (10F)	A (8×), H (2×)	KY962016, KY962017
		Srebrenik, Zvornik	red fox	*I. hexagonus* (2N)	U (2×)	KY962018
	Serbia	Svilajnac	red fox	*I. canisuga* (1F)	H	KY962030
		Boljevci	red fox	*I. canisuga* (1F, 1N)	A, H	KY962031, KY962030
		Progar	red fox	*I. canisuga* (1M)	A	KY962031
		Despotovac	red fox	*I. kaiseri* (1N)	N	KY962032
		Boljevci	red fox	*I. kaiseri* (5N)	L (5×)	KY962033
	Romania	Iazurile	dog	*I. kaiseri* (1F)	P	KY962020
		Cefa	red fox	*I. kaiseri* (1N)	P	KY962020
		Sǎlard	red fox	*I. kaiseri* (1F)	R	KY962024
		Popesti	red fox	*I. kaiseri* (2F, 1N)	P (2×), L	KY962020, KY962026
		Sǎnpetru	red fox	*I. kaiseri* (4F, 4N)	P (2×), L (4×), Q (2×)	KY962020, KY962026, KY962027
		Ilia	red fox	*I. kaiseri* (1F, 1N)	L, T	KY962026, KY962028
		Cefa	red fox	*I. canisuga* (2F, 1N)	A (2×), D	KY962021, KY962022
		Sǎtmǎrel	red fox	*I. canisuga* (2F)	D, K	KY962022, KY962023
		Cusuius	red fox	*I. canisuga* (1N)	C	KY962025
		Hodisel	red fox	*I. canisuga* (1F)	D	KY962022
		Ilia	red fox	*I. hexagonus* (1N)	U	KY962029

^a^Position of mutations in *cox*1 haplotypes (“-” = reference): *Ixodes canisuga*: A (-), B (57), C (144), D (291), E (441), F (498), G (525), H (37, 177), I (55, 525), J (37, 177, 363), K (37, 177, 453); *I. kaiseri*: L (-), M (60), N (444), O (60, 94), P (525, 588), Q (282, 525, 588), R (456, 525, 588), S (175, 330, 525, 588), T (234, 393, 525, 552, 588); *I. hexagonus*: U (-), V (366), W (442), X (366, 588), Y (66, 366, 588)

*Abbreviations*: *F* females, *M* males, *N* nymphs

**Table 2:** Haplotypes of 16S rRNA gene sequences according to the country of origin

Continental region	Country	*Ixodes* species (no. of samples analysed)	16S rRNA haplotypes^a^ marked with Roman numerals (number if >1)	GenBank accession nos.
Western Europe	UK	*I. canisuga* (2)	II (2)	KY962071
	France	*I. canisuga* (3)	I (2), II	KY962075, KY962074
Central Europe	Germany	*I. kaiseri* (2)	IV (2)	KY962067
		*I. canisuga* (2)	I, II	KY962068, KY962069
		*I. hexagonus* (1)	VII	KY962070
	Austria	*I. hexagonus* (2)	VII	KY962058
	Hungary	*I. kaiseri* (3)	IV, V (2)	KY962052, KY962054
		*I. canisuga* (2)	I (2)	KY962053
South-eastern Europe	Croatia	*I. hexagonus* (4)	VI (3), VII	KY962076, KY962077
		*I. canisuga* (3)	I (2), III	KY962072, KY962073
	Bosnia and Herzegovina	*I. canisuga* (2)	I, II	KY962055, KY962056
		*I. hexagonus* (1)	VI	KY962057
	Serbia	*I. canisuga* (2)	I, II	KY962065, KY962064
		*I. kaiseri* (2)	IV (2)	KY962066
	Romania	*I. kaiseri* (5)	IV (2), V (3)	KY962062, KY962059
		*I. canisuga* (4)	I (2), II^b^	KY962060, KY962061
		*I. hexagonus* (1)	VI	KY962063

^a^Position of mutations in 16S rRNA haplotypes (“-” = reference): *Ixodes canisuga*: I. (-), II. (178), III. (69); *I. kaiseri*: IV. (-), V. (215, 217); *I. hexagonus*: VI. (-), VII. (163)

^b^Sequencing of the 16S rRNA gene fragment from *cox*1 haplotype “C” was unsuccessful

For comparison and phylogenetic analyses, the sequences were trimmed to the same length (*cox*1: 631 bp, 16S rRNA gene: 402 bp). Tick species from other studies were included in the phylogenetic analyses only if their sequence(s) available in GenBank had 99–100% coverage with the sequences in this study. This dataset was resampled 1000 times to generate bootstrap values. Phylogenetic analyses were conducted with the Maximum Likelihood method by using MEGA version 6.0. The MEGA model selection method was applied to choose the appropriate model (GTR and Jukes-Cantor model for *cox*1 and 16S rRNA genes, respectively). The ratio of haplotypes between different geographical regions was compared by Fisher’s exact test (condition of significance: *P* < 0.05).

## Results

### Morphological identification of female ticks

Altogether 113 female ticks (all *Pholeoixodes* spp.) were compared morphologically with adequate descriptions, and, when available, with type material. For their species identification, the following simplified keys were compiled, taking into account distinctive cardinal criteria observed in the present study.

### Key to the females of *Pholeoixodes* spp. of carnivores in Europe

1a Basis capituli ending anteriorly as cone-like protuberance with lateral surface forming obtuse angles relative to longitudinal axis of hypostome ................................................................................................ 2

1b Basis capituli is not cone-like anteriorly ........................….................................................................... 4

**Fig. 2:**
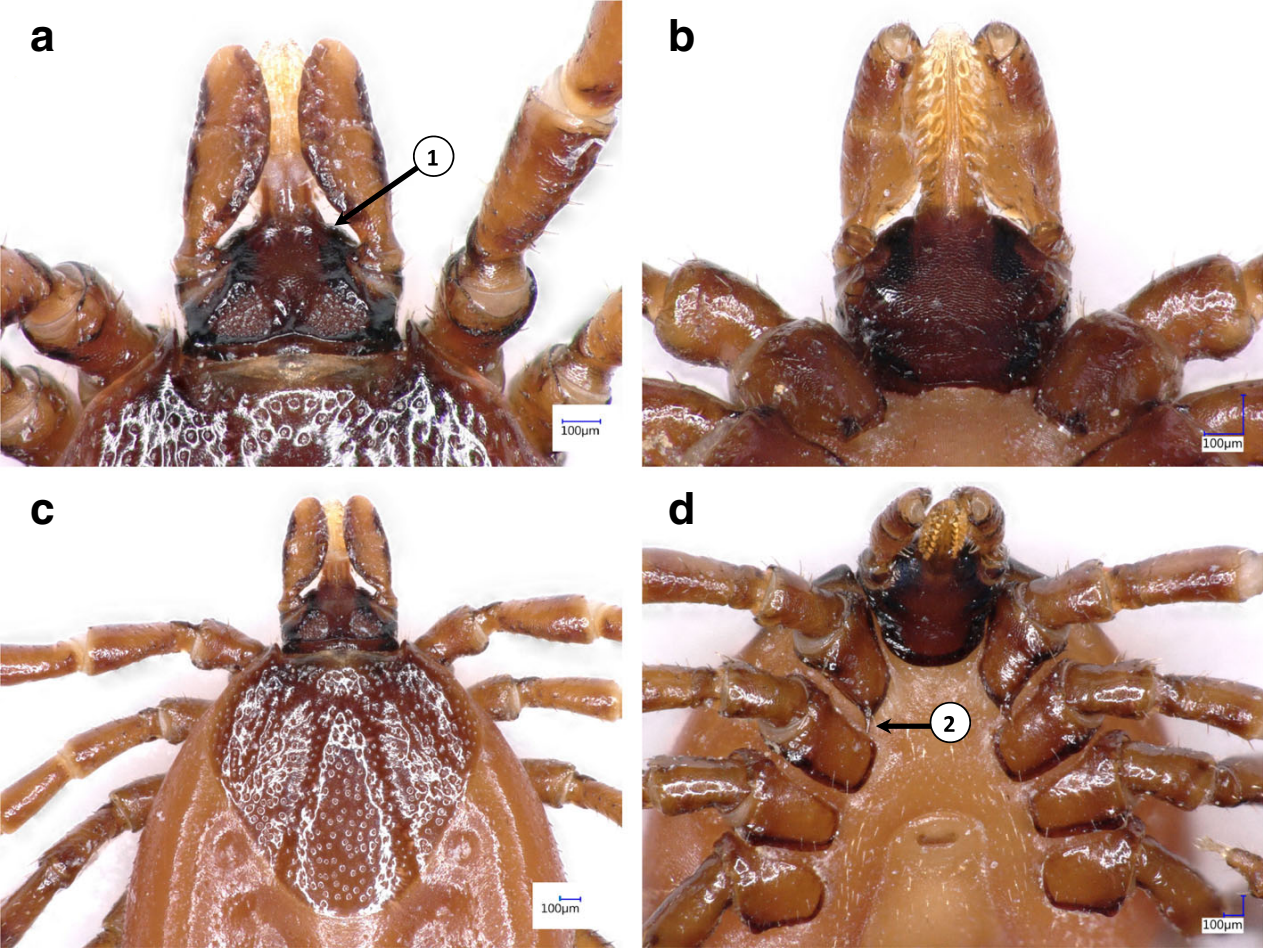
Key features of *Ixodes hexagonus* females. **a** Basis capituli, dorsal surface. **b** Basis capituli, ventral aspect. **c** Scutum and basis capituli. **d** Coxae I-IV. Numbered arrows indicate (in the order of presentation in the key): (1) cone-like anterior surface of basis capituli; (2) long and pointed internal spur on coxa I

2a Internal spur on coxa I long and pointed (Fig. 2) .......................................................................... *Ixodes hexagonus*

2b Internal spur on coxa I short and blunt ......................................................................................................... 3

**Fig. 3:**
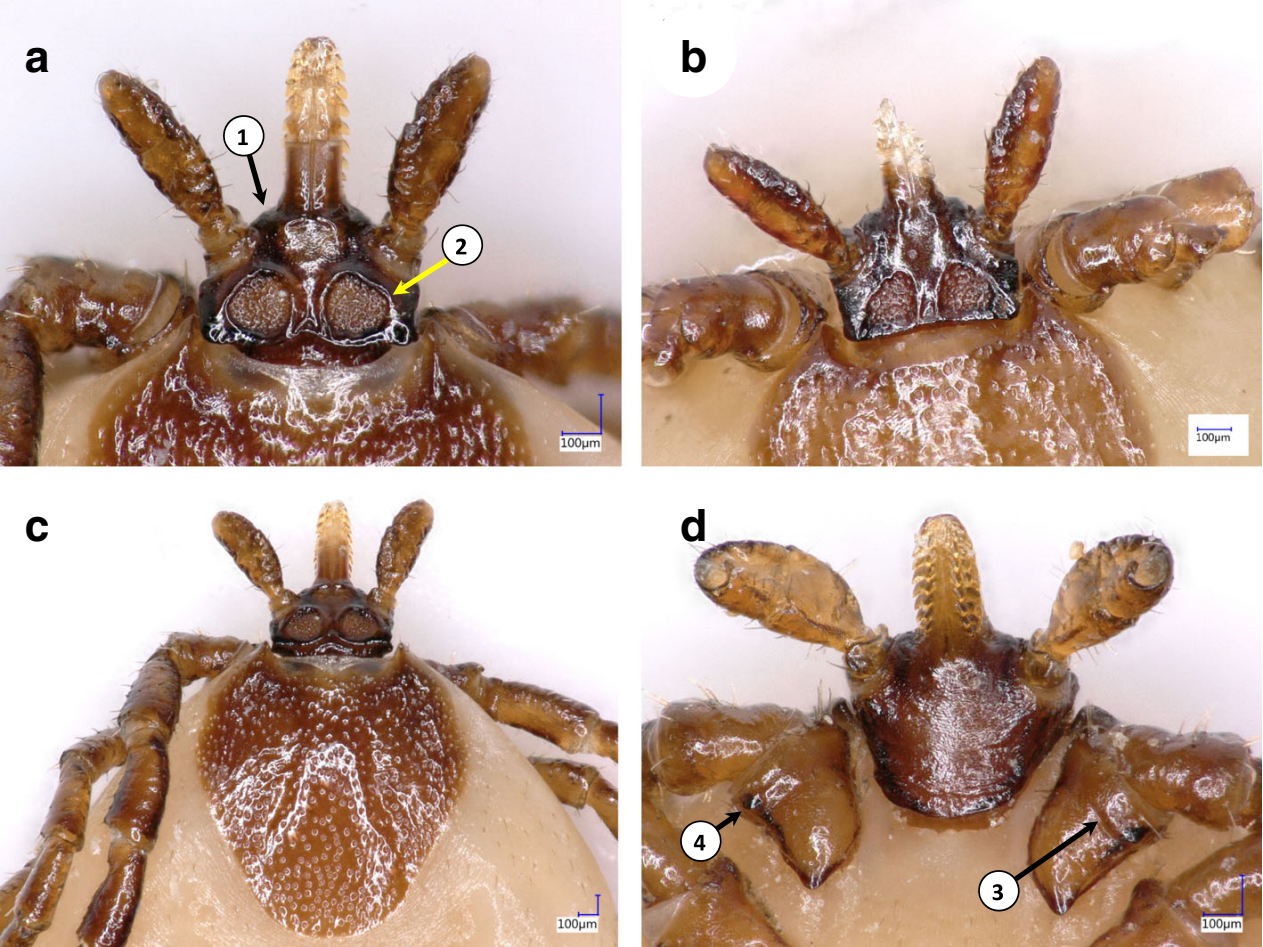
Key features of *Ixodes kaiseri* females. **a** Basis capituli, dorsal surface with rounded porose areas. **b** Basis capituli of another morphotype, with triangular porose areas. **c** Scutum and basis capituli. **d** Basis capituli, ventral aspect with coxae I. Numbered arrows indicate (in the order of presentation in the key): (1) cone-like shape of the anterior surface of basis capituli; (2) ridge-like margin of porose area; (3) longitudinal line (starting medially to the basis of external spur), which divides the surface of coxa I; (4) external spur on coxa I

3a Hypostome length to width ratio approx. 3:1; porose areas with ridge-like margins; surface of coxa I divided by a longitudinal line; external spurs as distinct small tuberosities present on all coxae (Fig. 3) ................................................................................. *Ixodes kaiseri*

3b Hypostome length to width ratio approx. 2:1; presence of double longitudinal ridges between the porose areas, diverging anteriorly towards anterolateral edges of basis capituli ........................................................................... *Ixodes crenulatus*

**Fig. 4:**
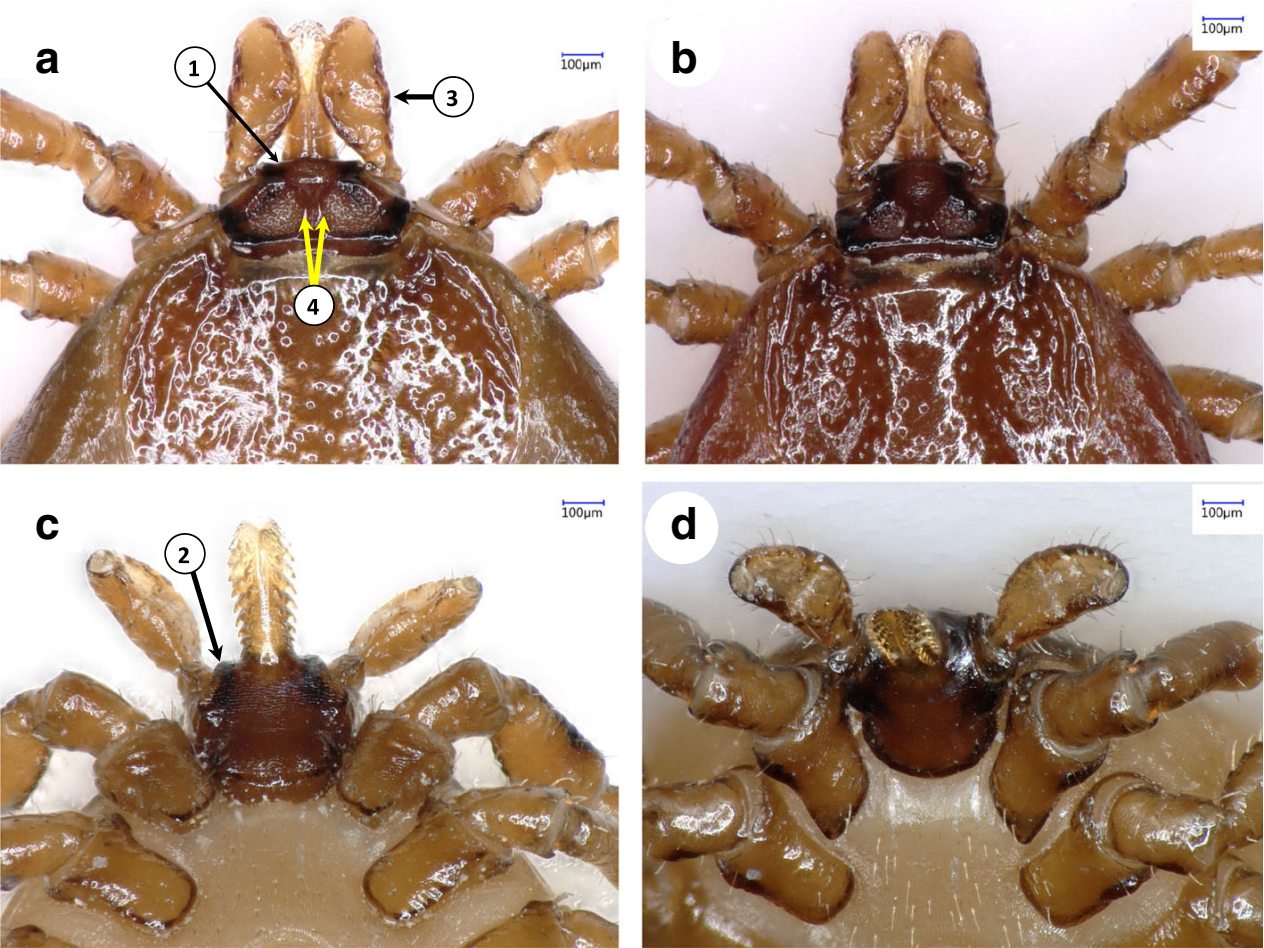
Key features of *Ixodes canisuga* females. **a** Basis capituli, dorsal surface. **b** Basis capituli of another morphotype, with considerably smaller porose areas. **c** Basis capituli, ventral aspect. **d** Coxa I (short, blunt internal spur viewed from a proper angle). Numbered arrows indicate (in the order of presentation in the key): (1) flat “plateau-like” anterior surface of basis capituli around the hypostome basis; (2) inconspicuous, rounded bump, i.e. slightly forward projecting ridge of “plateau”; (3) relatively straight lateral edge of palp; (4) narrow space between porose areas (i.e. less than their diameter)

**Fig. 5:**
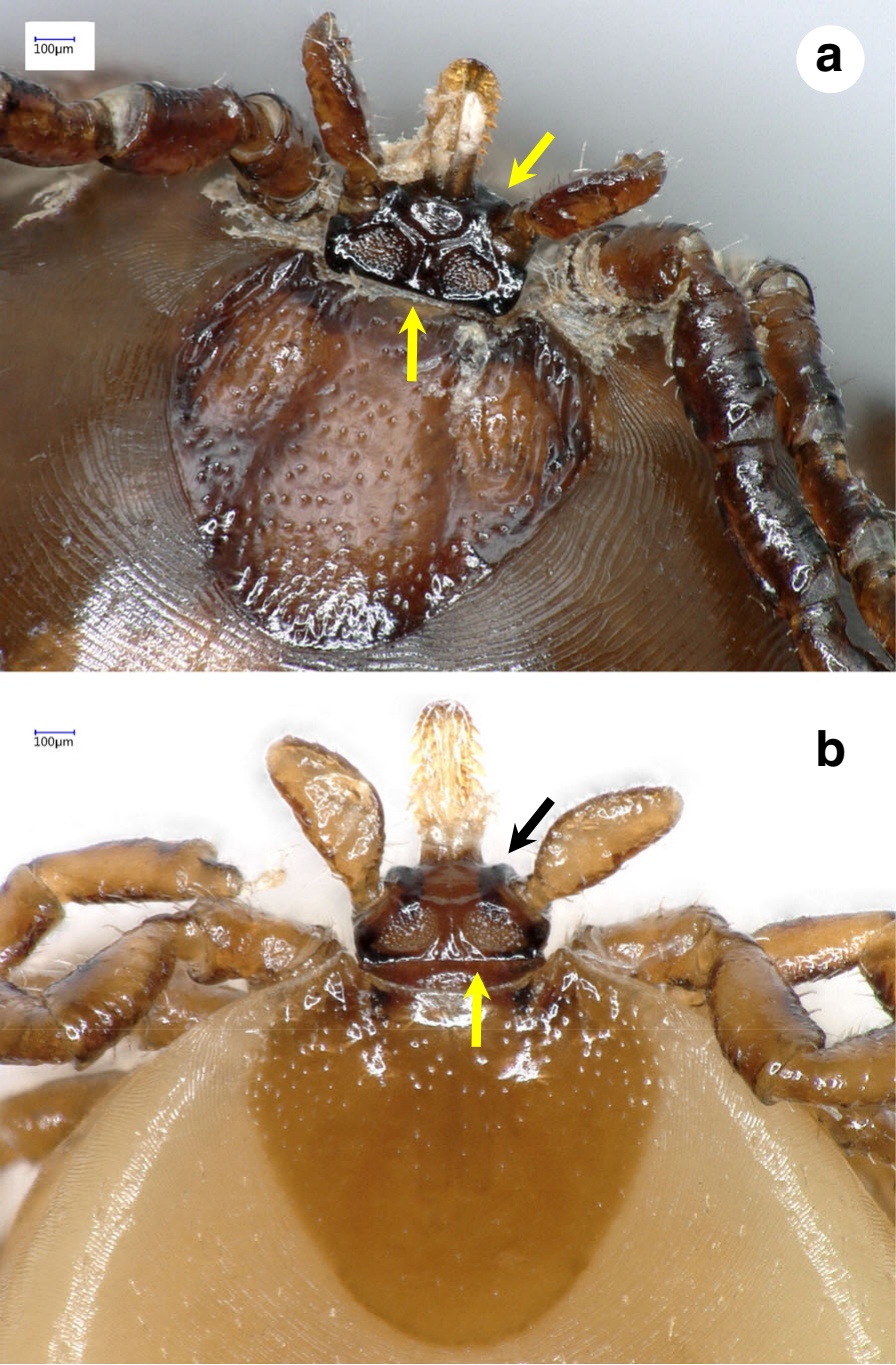
The similarity of *Ixodes canisuga* females to *I. crenulatus*. Basis capituli with dorsal ridge (between arrows) in case of two identical haplotypes (“A”) of *I. canisuga*, which thus resemble *I. crenulatus*. **a** Female from Bosnia and Herzegovina. **b** Female from Hungary

4a Anterior surface of basis capituli flat (plateau-like), perpendicular to longitudinal axis of hypostome, inconspicuous rounded bumps on anterior surface of basis capituli between hypostome and palps, palps laterally straight; separation of porose areas less than their diameter (Figs. 4, 5) ....................................................................................... *Ixodes canisuga*

4b Pronounced and pointed bumps on anterior surface of basis capituli between hypostome and palps, palps “stalked” and laterally curved (convex); space between porose areas more than twice as wide as diameter of porose area (Fig. 1) .......................................................... *Ixodes rugicollis*

By using these morphological keys, the material collected and further analyzed consisted of 71 females identified as *I. canisuga*, 21 females as *I. kaiseri* and 21 females as *I. hexagonus*. Neither *I. crenulatus* nor *I. rugicollis* were found.

### Molecular and phylogenetic analyses

DNA was extracted from 144 ticks (113 females, 30 nymphs, one male). Thus, 84 DNA samples of *I. canisuga*, 34 DNA samples of *I. kaiseri* and 26 DNA samples of *I. hexagonus* were analyzed. Ticks identified as *I. canisuga* had 11 (“A to K”) *cox*1 haplotypes: two of them were represented by seven and five individuals, respectively, while the remaining nine were found only once. These haplotypes showed up to three nucleotide differences from each other, corresponding to 99.5–100% sequence identity (628–631/631 bp). The distribution of some haplotypes reflected a clear geographical pattern (Table 1). Haplotype “H” was significantly more frequently identified in samples from western Europe than from central and south-eastern Europe (*P* < 0.0001). Several other haplotypes were identified only in one country (e.g. “B” in Hungary; “C”, “D” and “K” in Romania; “E”, “G” and “I” in Croatia; “F” in France; “J” in UK). However, haplotype “A” occurred in all evaluated regions of Europe (Table 1).

Ticks identified as *I. kaiseri* had nine (“L to T”) *cox*1 haplotypes, which showed a higher rate of polymorphism compared to *I. canisuga*, i.e. up to five nucleotide differences from each other (626–631/631 bp = 99.2–100% sequence identity). The occurrence of these haplotypes was restricted to central and south-eastern Europe (Table 1). Haplotypes “M” and “O” were unique to Germany, whereas the others occurred in Hungary and south-eastern Europe (Table 1). Ticks identified as *I. hexagonus* had five (“U to Y”) *cox*1 haplotypes, which showed up to three nucleotide differences from each other, i.e. 99.5–100% sequence identity (628–631/631 bp). Haplotype “X” was only identified in Germany and Austria, whereas all the others were present in south-eastern Europe (Table 1).

The 16S rRNA gene sequences of analyzed *Pholeoixodes* ticks had lower degree of intraspecific divergence compared to *cox*1 (i.e. 400–402/402 bp, i.e. 99.5–100% sequence identity) and fewer haplotypes (I–VII: Table 2). These haplotypes did not show geographical separation (e.g. haplotypes I-II occurred in western, central and south-eastern Europe).

**Fig. 6:**
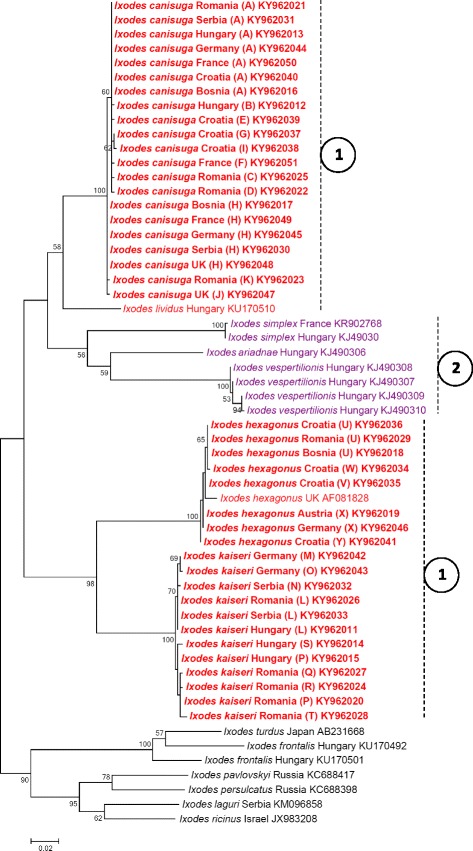
Phylogenetic tree based on the *cox*1 gene, including sequences obtained in this study (indicated with bold characters) and representative sequences of other *Ixodes* spp. from the GenBank. *Pholeoixodes* spp. are marked with red color and dashed vertical lines connected to encircled #1; *Eschatocephalus* spp. are marked with purple color and dashed vertical line connected to encircled #2. Between the species name and the accession number, the country of origin is shown. Branch lengths represent the number of substitutions per site inferred according to the scale shown

**Fig. 7:**
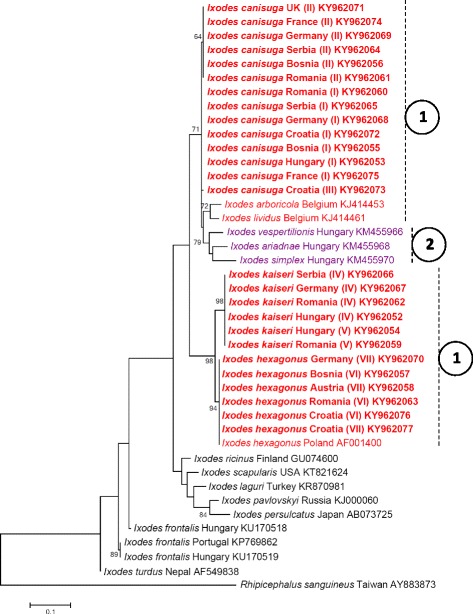
Phylogenetic tree based on the 16S rRNA gene, including sequences obtained in this study (indicated with bold characters) and representative sequences of other *Ixodes* spp. from the GenBank, as well as *Rhipicephalus sanguineus* as outgroup. *Pholeoixodes* spp. are marked with red color and dashed vertical lines connected to encircled #1; *Eschatocephalus* spp. are marked with purple color and dashed vertical line connected to encircled #2. Between the species name and the accession number, the country of origin is shown. Branch lengths represent the number of substitutions per site inferred according to the scale shown

The phylogenetic relationships of *cox*1 and 16S rRNA haplotypes are shown in Figs. 6 and 7, respectively. Morphological identification of the three species was supported by the phylogenetic analyses, because all morphologically a priori identified individuals of the three *Pholeoixodes* species grouped in the phylogenetic trees (Figs. 6, 7). The topologies of both phylogenetic trees reflect that (based on the investigated sequences) the subgenus *Pholeoixodes* is not monophyletic. Isolates of *I. kaiseri* and *I. hexagonus* formed two sister groups, whereas samples of *I. canisuga* were more closely related to the bat tick species *I. vespertilionis*, *I. ariadnae* and *I. simplex* (subgenus *Eschatocephalus*). Thus, the phylogenetic group of *Pholeoixodes* spp. also contained the clade of *Eschatocephalus* spp. (Figs. 6, 7).

## Discussion

This is the first comprehensive phylogenetic analysis involving three tick species from the subgenus *Pholeoixodes* collected from carnivores in Europe. In the present study, the morphological identification of *I. canisuga* and *I. hexagonus* was confirmed with molecular-phylogenetic methods, validating the morphological characters selected and described in the above taxonomic key. This identification key highlights differences among *Pholeoixodes* spp. in the shape of the anterior surface of basis capituli (between the hypostome and the palps). While the shape of the anterior surface of basis capituli has been reported to be a distinctive character between various *Ixodes* spp., including *I. canisuga* and *I. hexagonus* [32], this character has not been incorporated into the identification keys provided by other authors (e.g. [20]). These data suggest that the shape of the anterior surface of basis capituli should always be observed for the morphological differentiation of female *Pholeoixodes* ticks of carnivores (i.e. *I. canisuga* *vs* *I. hexagonus* and *I. kaiseri*).

The data presented here expand the known geographical range of *I. kaiseri* in Europe. Recently, ticks resembling *I. kaiseri* were reported in Poland [33]. Although *I. kaiseri* was previously reported from Romania [34], this was not confirmed by later studies involving or reporting ticks of carnivores [8, 35–37]. Here, the evidence is provided for the occurrence of *I. kaiseri* in Romania, as well as in three other countries, where it had not been reported yet (Germany, Hungary and Serbia). These new records are probably not a consequence of recent emergence of this tick species in new regions, but rather that its specimens have hitherto been misdiagnosed. Therefore, the keys presented here will be useful for future studies, which will help to define the geographical range of *I. kaiseri*.

*Ixodes crenulatus* was not found in the present study, not even in Romania, where it has been recently reported to occur [8]. On the other hand, reports of its occurrence in western Europe (Ireland, UK, Germany) are more than half a century old [7], and require verification. The diagnosis of this species is difficult, because the redescription (although adequate) is not easily accessible and is written in Russian [14], and no type-specimen is available [2]. The most important diagnostic feature of *I. crenulatus* females, the longitudinal ridges on the basis capituli [14] bear a resemblance to the ridge (rounded bumps) of the plateau seen in *I. canisuga* (Fig. 5), therefore further studies involving both species will be needed to reassess their synonymy, which was proposed by some authors [2].

Similarly, based on the inadequacy (and contradictions) of descriptions, illustrations in some former and recent reports on *I. rugicollis*, the relevant data should be interpreted with caution, taking into account the type specimens and key of females presented here (in Romania [7]: porose areas different, frontal bumps absent; in Poland [38]: porose areas not shown, frontal bumps are rounded). Also, *I. rugicollis* has recently been identified in Hungary [39] in part according to the description of *I. cornutus* [14], which was regarded as a synonym of *I. rugicollis* [26]. However, based on the type specimens investigated here and the identification criteria presented in the key, this synonymy cannot be maintained.

Phylogenetic analyses performed here suggest that the subgenus *Pholeoixodes* is not monophyletic. Taking into account that in both the *cox*1 and 16S rRNA gene phylogenetic analyses *I. canisuga* specimens (and *I./Ph. lividus*) formed one clade with bat ticks of the subgenus *Eschatocephalus*, it is necessary to test the parsimony of the inclusion of the latter subgenus in *Pholeoixodes*. Alternatively, *Pholeoixodes* should be divided into two subgenera. These data, on the other hand, confirm the phylogenetic relevance of morphological traits, because these bat tick species lack auriculae and long/pointed internal spur on coxa I, similarly to most *Pholeoixodes* spp.

The results presented here should also be considered in a geographical context. Although geographical structuring of several (but not all) *cox*1 mitochondrial lineages was observed, sequence and phylogenetic analyses of the 16S rRNA gene did not reflect the same pattern. These findings suggest that genetic exchange within each *Pholeoixodes* species is not limited between different European populations investigated here, i.e. these tick species are not subdivided into geographically distinct populations, but undergo constant gene flow. One underlying reason may be that populations of an important host species, the red fox (*Vulpes vulpes*) are apparently in contact and mix throughout Europe, allowing gene flow, with little spatial structuring [40]. The emergence of golden jackals (*Canis aureus*) towards western Europe [41] may have also contributed to the dispersal of tick species investigated here. This could also counterbalance genetic differences that would have otherwise resulted from separation of populations of the main hosts (hedgehog species) of *I. hexagonus* (i.e. *Erinaceus europaeus* in Germany *vs* *E. roumanicus* in Hungary and south-eastern Europe).

## Conclusions

Reference sequences are provided for *I. canisuga* and *I. kaiseri* based on female specimens determined according to simple identification keys (including *I. hexagonus* and *I. rugicollis*), to prevent future misdiagnoses of these species. These results confirm that the shape and the morphological features of the anterior surface of basis capituli, the details of spurs on coxa I and the relative width of the scutum of females are important characters for species identification among *Pholeoixodes* ticks of carnivores. It is also demonstrated that *I. kaiseri* is more widespread in Europe than previously thought. Based on phylogenetic analyses, the subgenus *Pholeoixodes* is not monophyletic, as the subgenus *Eschatocephalus* clustered within its clade. This should be further elaborated by future taxonomic studies.

## Abbreviations

NHM: The Natural History Museum, London, UK
USNTC: United States National Ticks Collection, Statesboro, USA
